# Colorectal cancer – patterns of locoregional recurrence and distant metastases as demonstrated by FDG PET / CT

**DOI:** 10.4103/0971-3026.73545

**Published:** 2010-11

**Authors:** Nilendu C Purandare, Sumeet G Dua, Abhishek Arora, Sneha Shah, Venkatesh Rangarajan

**Affiliations:** Bio-Imaging Unit, Tata Memorial Hospital, Dr. Ernest Borges Marg, Parel, Mumbai- 400 012, India

**Keywords:** Fluorodeoxyglucose (FDG) PET / CT, colorectal cancer, recurrence disease

## Abstract

Colorectal cancer (CRC) can recur locoregionally or at distant sites. Timely diagnosis of recurrence is of paramount importance, as radical treatment of the localized disease can prolong survival. Fluorodeoxyglucose positron emission tomography / computed tomography (PET / CT) is routinely used in restaging and surveillance of colorectal cancer, as it can demonstrate recurrent disease with good accuracy. This article illustrates the spectrum of standard as well as unusual patterns of local recurrence and distant metastases of colorectal cancer.

## Introduction

Recurrence of colorectal cancer (CRC) is seen in about 30 – 40% of patients who undergo primary curative surgical resection. The majority of these recurrences occur in the first two years after surgery.[1] Radical treatment of isolated local recurrences and hepatic and pulmonary metastases has been shown to improve survival;[2,3] however, such interventions, in the presence of metastases at other sites, have failed to result in significant survival advantage.[4] Hence, imaging can play a very important role in detecting early recurrent disease, while the recurrence is still localized and resectable. Delbeke and colleagues[5] have shown that fluorodeoxyglucose PET (FDG-PET) can detect occult metastases in 32% of the patients, and thereby change the course of treatment in more than one-fourth of the cases. In addition, the role of FDG PET / CT as a problem-solving tool in patients on follow-up for a treated CRC, has been increasing in the setting of unexplained elevation of carcinoembryonic antigen and equivocal findings on conventional imaging modalities.[6] In this pictorial essay, we illustrate the spectrum of recurrence of CRC and the role of FDG PET / CT in its detection, characterization, and treatment response evaluation.

### Local or locoregional recurrence

Pelvic recurrence in operated CRC occurs in as much as 30% of the cases. Recurrence may be seen in the pelvic nodes, at the anastomotic site or rectal stump, or in the presacral area, as a soft tissue mass. The importance of early detection of local recurrence at an operable stage cannot be overemphasized, in view of literature reports showing improved survival following resection of localized recurrences.[7]

### Presacral and pelvic soft tissue recurrence

**Figure 1 (A,B):**
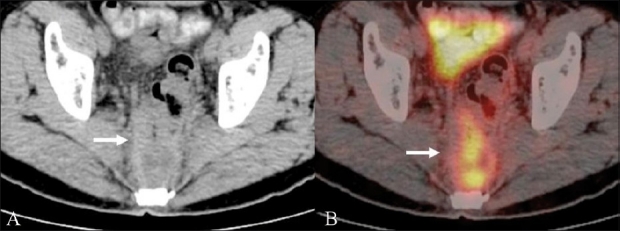
A 57-year-old man with a history of abdominoperineal resection (APR) for rectal cancer presented with symptoms of pelvic pain and underwent a restaging FDG PET / CT. Axial unenhanced CT scan (A) shows a heterogeneous soft tissue mass in the presacral region (arrow). Fusion PET / CT image (B) reveals increased FDG uptake within the soft tissue (arrow), suggestive of local recurrence

**Figure 2 (A,B):**
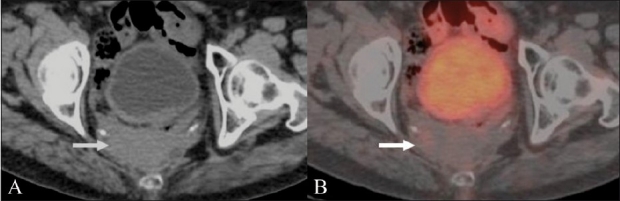
A 48-year-old woman with rectal cancer treated with APR and radiation therapy underwent a restaging FDG PET / CT for suspected recurrence. Axial unenhanced CT scan (A) shows a soft tissue mass in the presacral region (arrow). Fusion PET / CT image (B) shows no FDG concentration within the soft tissue (arrow), suggesting post-treatment fibrosis

**Figure 3 (A,B):**
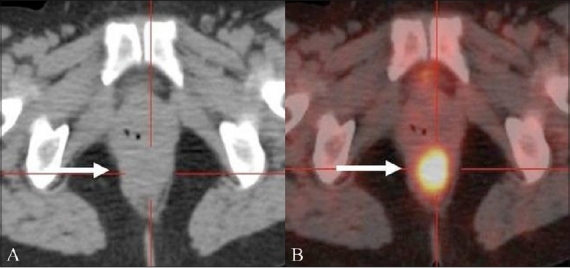
A 60-year-old man treated with surgery for low rectal cancer presented with bleeding per rectum and underwent an FDG PET / CT study. Axial unenhanced CT scan (A) shows ill-defined soft tissue thickening in the perianal region (arrow) without definite evidence of a nodule / mass. Fusion PET / CT image (B) shows an intense focus of hypermetabolism corresponding to the soft tissue thickening, suggesting perianal recurrence

Surgery for CRC and radiation therapy-associated inflammatory changes can lead to both anatomic distortion in the pelvis, and often predispose to the development of a fibrotic presacral mass. This can occur in as many as 39% of the patients following anterior resection and in 19% of the patients after abdominoperineal resection.[8] CT scanning, due to its reliance on size and morphology, has limitations differentiating between fibrotic masses and soft tissue recurrences. On FDG-PET imaging, a recurrent mass in the presacral region shows increased tracer uptake, thus differentiating it from the fibrotic tissue [Figures 1 and 2]. PET / CT has shown higher accuracy when compared to PET and CT scan individually in differentiating a fibrotic presacral mass from recurrent disease.[9] Although FDG uptake shows disease in CT scan negative areas, a CT scan often solves the problem of physiological FDG uptake in normal structures such as the urinary bladder and bowel, which prolapse into the empty rectal fossa and may give rise to false positive PET results. In the perianal and rectal stump regions, however, the CT scan has limited contrast resolution and FDG-PET can be very useful in picking up subtle recurrence [Figure 3].

### Anastomotic site recurrences

**Figure 4 (A,B):**
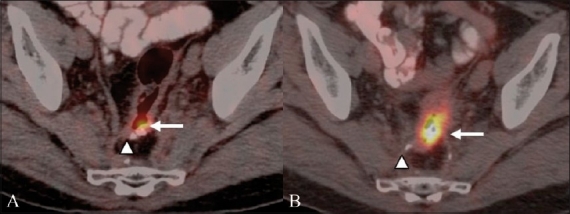
A 67-year-old man operated for rectosigmoid malignancy presented with rising tumor marker levels and underwent an FDG PET / CT study. The fusion FDG PET / CT image (A) reveals a tiny, but intense focus of FDG uptake (arrow) at the rectosigmoid anastomotic site. A contrast-enhanced CT scan of the abdomen and pelvis and colonoscopy, however, did not reveal recurrence; hence, the patient was kept under observation. A follow-up PET / CT study done after eight weeks (B) shows disease progression, by demonstrating increase in the extent and intensity of the FDG uptake, with the appearance of a soft tissue mass at the anastomotic site. A colonoscopic biopsy confirmed recurrence. Hyperdense surgical staples (arrowheads in A and B) mark the anastomotic site.

Recurrence at the anastomotic site is often encountered. Resection of such localized recurrences offers a survival advantage. The typical PET / CT appearance is of a hypermetabolic soft tissue mass or subtle wall thickening at the anastomotic site,[10] which is often identified by a surgical ring of radio-opaque staples [Figure 4]. Although colonoscopy would be the ideal technique for diagnosing and confirming anastomotic site recurrences, FDG PET / CT imaging can prove to be an excellent noninvasive modality when such recurrences are suspected.

### Pelvic nodal recurrence

**Figure 5 (A,B):**
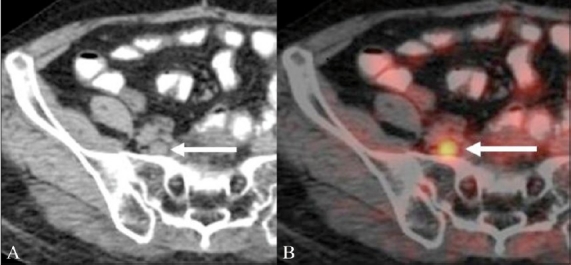
A 62-year-old man treated for CRC was imaged with FDG PET / CT during routine surveillance. An axial unenhanced CT scan (A) reveals a sub-centimeter-sized, round, right common iliac node (arrow) that shows increased FDG uptake (arrow) on the fusion PET / CT image (B). USG-guided fine needle biopsy confirmed nodal recurrence

Categorization of nodes as metastatic on conventional imaging modalities, including CT scan and magnetic resonance imaging (MRI), is based on their size. This approach results in a decrease in the reporting of metastatic recurrence in centimeter-sized nodes, which are often seen in CRC. By virtue of its ability to superimpose metabolic information on the anatomic detail, the PET / CT helps characterize even centimeter-sized metastatic nodes [Figure 5], with a resultant reduction in false negative restaging studies.[11]

### Distant metastases

#### Liver and lung metastases

Resection of operable hepatic and pulmonary metastases offers the only chance of cure and serves to prolong survival in CRC; however, the presence of extrahepatic or extrapulmonary metastases is associated with poor survival despite metastatectomy. According to a recent prospective multicenter study, which evaluated the role of FDG-PET in recurrent colorectal cancer, 23.5% of the patients with potentially resectable hepatic and pulmonary metastases, on conventional imaging, were deemed inoperable as a result of their PET scan findings.[12] As mentioned earlier, the PET / CT can detect occult metastases in about one-third of the patients with CRC, and thereby alter the management.[13]

**Figure 6 (A,B):**
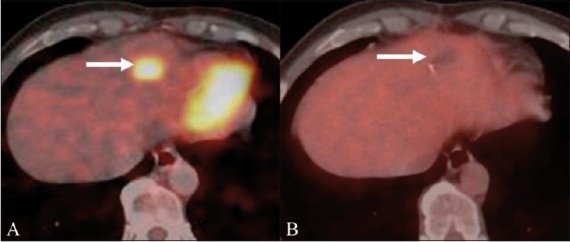
FDG PET / CT evaluation of a solitary hepatic metastasis in an operated case of colon cancer, treated with radiofrequency ablation (RFA). Pre-RFA fused PET / CT (A) image shows an FDG-avid metastasis in the left lobe of the liver (arrow). Immediate post-RFA fused PET / CT (B) image shows complete photopenia at the ablated site (arrow), suggesting complete ablation

In a meta-analysis, when comparing FDG-PET, CT scan, and 1.5 T MRI in colorectal liver metastases, FDG was found to be the most accurate modality on a per-patient basis, whereas, the modalities were more or less comparable on a per-lesion basis.[14] However, the MRI using liver-specific contrast agents was found to be superior to FDG-PET in the detection of small liver metastases.[15] In addition, PET / CT also showed significantly higher specificity (100%) than the contrast-enhanced CT scan (50%) in the detection of recurrences following hepatic resection,[16] as well as following radiofrequency ablation (RFA).[17] Complete photopenia at the ablated site on the FDG-PET scan suggested a metabolic response and completeness of the ablation[18,19][Figure 6].

#### Peritoneal deposits

**Figure 7 (A,B):**
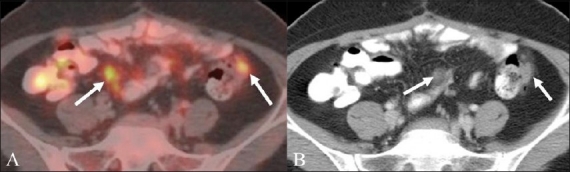
A 39-year-old woman with a history of CRC. Axial fusion PET / CT (A) image shows hypermetabolic FDG-avid nodular foci abutting the bowel surface. Axial contrast-enhanced CT scan at the same anatomical level (B) shows enhancing nodular serosal deposits along the bowel surface (arrows) corresponding to the hypermetabolic foci in (A). Note the striking conspicuity of the deposits in (A) as compared to the subtle findings in (B)

**Figure 8 (A-C):**
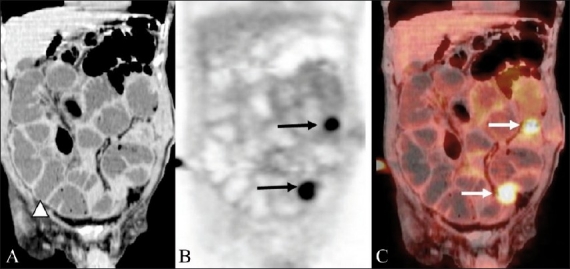
A 52-year-old man treated for CRC presented with abdominal distention and vomiting. Coronal unenhanced CT scan (A) shows multiple dilated small bowel loops (arrowhead). The coronal PET image (B) reveals discrete foci of abnormal FDG uptake in the abdominal cavity (arrows). These foci were mapped to the peritoneal surface of the small bowel (arrow) on the fusion PET / CT image (C), suggesting metastatic peritoneal implants as the cause of intestinal obstruction

The development of peritoneal disease [Figures 7 and 8] in the setting of CRC carries a grave prognosis, with little — if any — response to surgical therapy or chemotherapeutic agents.[20] The prognosis is slightly better in the localized foci of peritoneal disease, as they are amenable to complete resection and thus call for accurate and timely detection.[20,21] Occasionally these deposits can result in bowel adhesion and consequent intestinal obstruction [Figure 8].

### Abdominal wall and colostomy site recurrence

**Figure 9 (A,B):**
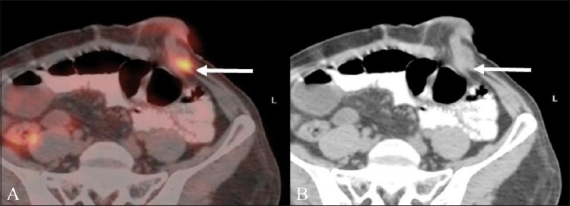
A 59-year-old man who underwent an abdominoperineal resection for low rectal cancer came for follow-up. An FDG PET / CT image (A) reveals a focus of abnormal FDG uptake at the colostomy site (arrow). Close inspection of the CT scan (B) reveals an inconspicuous soft tissue nodule (arrow) corresponding to the increased FDG uptake, suggesting recurrence at the colostomy site. Postoperative changes in the anterior abdominal wall can often mask such recurrent nodules, which are detected on PET, by virtue of their hypermetabolism.

Scars from open or laparoscopic surgery, as well as drain, port, and stoma sites, are potential locations of metastatic CRC recurrence [Figure 9]. Resection of such metastases can be considered in the absence of disease in the abdomen or elsewhere as it can result in adequate local control with minimal procedural complications.[22,23] PET / CT is a sensitive tool in the detection of abdominal wall, stoma / port-site metastases of CRC.[24]

### Infrequent sites

**Figure 10 (A-F):**
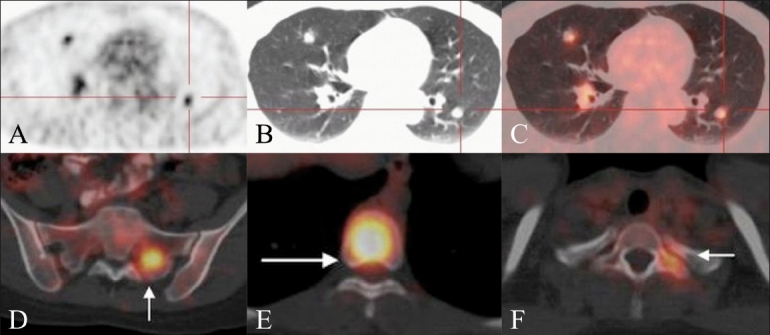
Restaging FDG PET / CT studies performed on two different patients of treated CRC. Axial PET (A), axial CT scan (B), and fused axial PET / CT (C) images show multiple FDG-avid lung metastases. Axial fused PET / CT images of another patient of CRC show FDGavid skeletal metastases in the left sacral ala (arrow in D), D8 vertebral body (arrow in E), and in the left transverse process of the D1 vertebra (arrow in F)

**Figure 11 (A-C):**
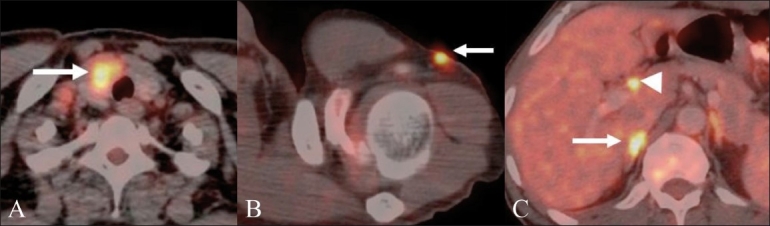
Restaging FDG PET / CT studies performed on three different patients of treated CRC for suspicion of disease recurrence show unusual metastatic sites; in the right lobe of the thyroid gland (arrow in A), in the subcutaneous region of the left upper arm (arrow in B), metastatic portal adenopathy (arrowhead in c), and a right adrenal metastasis (arrow in C)

The incidence of metastases to infrequent sites is increasing due to the improved survival of patients. Skeletal [Figure 10D–F] and brain metastases are more likely to occur in the setting of lung metastases, [Figure 10A–C] and in a primary rectal cancer as compared to colon cancer.[25] Occasional cases of metastases from CRC to thyroid,[26] adrenals,[27] and subcutaneous tissues[28] [Figure 11] have also been documented in literature.

### FDG-PET in monitoring response to systemic therapy

**Figure 12 (A,B):**
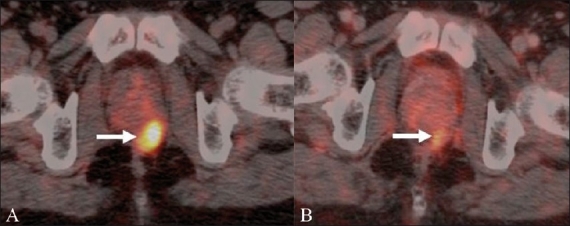
A 52-year-old man with metastatic CRC imaged with FDG PET / CT before and after targeted therapy. Fusion PET / CT study (A) prior to targeted therapy shows an FDG-avid metastatic retroprostatic nodule (arrow). There is significant reduction in the metabolism and size of the recurrent nodule (arrow) on the post-therapy follow-up PET / CT image (B), suggesting therapeutic response

Morphological imaging techniques are limited in assessing therapeutic response, as they rely on changes in tumor size, which often lag behind biological response. FDG-PET has been used to monitor early response to primary as well as secondary chemotherapeutic agents after the first or the second cycle in advanced CRC[29] [Figure 12]. Furthermore, with the advent of newer molecular targeted therapies that often target biological effects like angiogenesis, more accurate surrogate endpoints are required, to assess therapeutic response. FDG PET / CT can be very useful in this regard to identify responders early in the course of therapy.
